# Pakistan’s HPV vaccination drive: navigating trust, culture, and misinformation in a new era of immunization

**DOI:** 10.3389/ijph.2026.1609244

**Published:** 2026-06-11

**Authors:** Umaima Mir, Maria Asghar, Nayab Amjad, Urooj Amjad, Iqra Khan, Shandana Kifayat, Shanlina Kifayat, Muhammad Ali Jan Khan, Khadija Tul Kobra, Zeeshan Wali, Baber Wali, Sobia Shafiq, Asad Ullah, Fazal Jamil, Muhammad Ittefaq, Muhammad Salar Khan

**Affiliations:** 1 Lady Reading Hospital, Peshawar, Pakistan; 2 Mardan Medical Complex, Mardan, Pakistan; 3 Care and Curing Center, Peshawar, Pakistan; 4 Al Nafees Medical College, Islamabad, Pakistan; 5 Khyber Teaching Hospital, Khyber Medical University, Peshawar, Pakistan; 6 College of Physical Medicine and Rehabilitation Paraplegic Center, Khyber Medical University, Peshawar, Pakistan; 7 Northwest School of Medicine, Peshawar, Pakistan; 8 Ayub Medical College, Abbottabad, Pakistan; 9 Rehman Medical College, Peshawar, Pakistan; 10 Peshawar Dental College, Peshawar, Pakistan; 11 Hamdard Medical College, Karachi, Pakistan; 12 St. Elizabeth Youngstown Hospital, Youngstown, OH, United States; 13 Michigan State University, East Lansing, MI, United States; 14 Rochester Institute of Technology, Rochester, NY, United States

**Keywords:** HPV vaccination, vaccine hesitancy, trust and culture, policy, Pakistan public health

## Highlights

- Human papillomavirus vaccination prevents cervical cancer, but in Pakistan early roll-out shows uneven coverage driven by trust, stigma, and misinformation rather than supply alone.
- Partnering with respected religious leaders and community health workers can legitimize vaccination as cancer prevention aligned with family and faith.
- Proactive social listening and rapid rebuttals are needed to counter rumors before they spread on WhatsApp and other platforms.
- Reaching out-of-school girls and poorer districts -- free at point of care and embedded in the national immunization program -- is essential for equity and public confidence.

Cervical cancer is among the most preventable types of cancer, yet it remains a major cause of mortality in low- and middle-income countries (LMICs). Globally, it accounts for more than 340,000 deaths each year, with nearly 90% occurring in LMICs [1]. In Pakistan, cervical cancer ranks among the most common cancers in women, with an estimated 4,762 new cases and 3,069 deaths annually [2]. Although the human papillomavirus (HPV) vaccine is a safe and effective means of prevention, uptake in South Asia remains dismally low. A 2024 meta-analysis reported average coverage across the region of only about 8%, with Pakistan at the lower end [3]. In this Commentary, we propose immediate, practical measures to build trust to improve HPV vaccination uptake among eligible adolescent girls (9–14 years) in Pakistan.

In September 2025, Pakistan launched its first nationwide HPV vaccination campaign, aiming to immunize 13 million girls aged 9–14 years, with training for nearly 50,000 health workers and with plans to integrate the vaccine into the Expanded Program on Immunization (EPI) [4]. This campaign represents a historic step and the beginning of a new era of immunization in Pakistan, one that extends beyond infancy to include adolescent girls. Yet its success is far from guaranteed. Early reports show uneven coverage and parental skepticism, including markedly low uptake even in cities such as Karachi, and shortfalls relative to initial provincial targets [5]. Overall success will depend not only on logistics and supply but also on how effectively the program navigates cultural sensitivities, religious discourse, the spread of misinformation, and the legacies of past vaccination efforts.

The novelty of this campaign lies in its focus on adolescent girls, a group rarely targeted in Pakistan’s immunization landscape. Unlike infant vaccines, HPV requires parental consent for older children and inevitably touches on issues of sexuality, morality, and cultural values. This increases the risk of parental hesitancy, particularly in contexts where sexual and reproductive health is taboo. At the same time, the campaign is unfolding against a fraught history of vaccination. Polio eradication efforts in Pakistan have faced decades of setbacks, marked by refusals, entrenched rumor networks, and targeted violence, including attacks on female polio vaccinators and their security escorts [6]. A past incident in 2011 in which a health campaign was co-opted for non-health purposes intensified public skepticism toward vaccination drives [7]. Such events continue to shape how communities perceive door-to-door health programs.

COVID-19 vaccination added new complications but also provided lessons. Surveys across Pakistan revealed early hesitancy driven by fears about safety, efficacy, misinformation, and conspiracy theories [8]. These concerns mirror those now emerging around HPV. Yet COVID-19 uptake eventually increased, in part due to mandates, visible endorsements from political and religious leaders, and widespread availability in public facilities. Notably, national religious authorities publicly declared COVID-19 vaccination permissible under Sharia, illustrating how religious guidance can unlock acceptance when biomedical messaging alone stalls. The lesson is clear: trust is fragile but can be rebuilt through deliberate community engagement.

HPV vaccination, however, introduces a new layer of complexity. Because HPV is sexually transmitted, the vaccine is vulnerable to stigma and misinterpretation. Parents may worry that vaccination tacitly condones premarital sexual activity or even jeopardizes fertility. A recent systematic review of studies in Muslim-majority countries, including Pakistan, found that HPV vaccine hesitancy is shaped not only by doubts about safety and necessity but also by religious objections and moral anxieties, including fears of infertility, sexual promiscuity, and the presence of ‘haram’ ingredients [9]. If presented narrowly as a tool against infection, the vaccine may be rejected on moral grounds; reframing it as a cancer prevention measure, however, situates it as an act of parental protection and a religious duty to preserve life.

Religious authority is therefore not a barrier but a resource. During both polio and COVID-19 campaigns, religious scholars and imams issued public statements affirming the permissibility of vaccination under *Sharia* law. In high-risk areas of Karachi, parents themselves recommended that trusted figures such as well-known doctors, religious leaders, and sports or media personalities should be engaged as advocates for vaccination [6]. For HPV, early and sustained engagement with religious leaders is essential, not as token endorsement but as genuine partnership in message development. This engagement must emphasize the alignment between HPV vaccination and religious values of safeguarding health and family.

The battle over trust will not be fought only in mosques or clinics but also on smartphones. Rumors about infertility, Western plots, and “haram ingredients” spread rapidly on WhatsApp and Facebook. Experience from polio programming shows that waiting for rumors to crest is costly; proactive social listening, rapid rebuttals, and feedback loops can protect credibility before narratives harden.

Equity is equally decisive. Until recently, HPV vaccines were available almost exclusively in urban private hospitals, reinforcing perceptions of elitism and inaccessibility. The national campaign seeks to change this, but structural challenges remain. Almost 50% of girls in the target age group in Pakistan are not enrolled in school, meaning school-based campaigns alone will not achieve universal coverage or increase vaccine uptake. Outreach to slums, rural districts, and marginalized populations in provinces such as Sindh and Khyber Pakhtunkhwa is critical. Pilot campaigns have attempted to include out-of-school girls through neighborhood vaccination sites, but these must be scaled and sustained beyond one-off drives. While the current campaign, supported by Gavi, WHO, UNICEF, and other partners, is providing the HPV vaccine free of charge, its sustainability beyond external funding is uncertain. Embedding HPV vaccination within the EPI as a routine, government-financed service will be essential to maintain universal access. Without this long-term commitment, the vaccine risks again being seen as an intervention for elites, undermining trust and legitimacy.

Several pathways could help Pakistan navigate this moment. The Lady Health Worker program is uniquely positioned to build confidence, as these women are trusted household-level providers. Their proximity to families allows them to address hesitancy in ways that distant officials cannot. Religious scholars should be embedded as co-developers of messaging, allowing imams to frame vaccination as an act of parental responsibility rather than moral threat. Behavioral nudges should be incorporated: a recent mobile-based audio drama campaign in Pakistan led to a 30% increase in full childhood immunization in target areas relative to comparison areas, demonstrating how culturally tailored digital tools can shift behavior [10]. Similar strategies, including text reminders, school events, peer testimonials, could help normalize HPV vaccination. Finally, the government must expand beyond schools and urban centers, ensuring outreach in informal settlements, rural districts, and disaster-affected areas.

Although Pakistan’s HPV rollout is unique in timing and scale, its challenges are not unique. Across LMICs, immunization is shaped not only by technical logistics but also by culture, religion, and trust. In sub-Saharan Africa, Latin America, and the Middle East, HPV campaigns face similar barriers of stigma, misinformation, and inequitable access. Pakistan’s campaign therefore carries lessons with resonance beyond its borders. If the program succeeds in normalizing HPV vaccination in a conservative context, it can provide an example for other countries confronting similar obstacles. If it falters, the consequences may extend beyond Pakistan, reinforcing skepticism in comparable settings.

Pakistan’s HPV vaccination campaign currently as it stands is both historic and fragile. Its outcome will depend as much on relationships of trust as on syringes and cold chains. By embedding the program within trusted community networks, reframing the vaccine as cancer prevention, drawing lessons from past vaccination efforts, and ensuring equitable access, Pakistan has the opportunity to show that even the most culturally sensitive vaccines can succeed in conservative, resource-limited contexts. As health professionals, researchers, and educators with ties to both Pakistan and its diaspora, we write with a shared conviction that the world should not only observe but learn, global immunization equity depends not just on delivering vaccines, but on delivering them with dignity and trust.
